# Proteomic dissection of vanishing white matter pathogenesis

**DOI:** 10.1007/s00018-024-05258-4

**Published:** 2024-05-24

**Authors:** Jodie H. K. Man, Parand Zarekiani, Peter Mosen, Mike de Kok, Donna O. Debets, Marjolein Breur, Maarten Altelaar, Marjo S. van der Knaap, Marianna Bugiani

**Affiliations:** 1 Department of Child Neurology, Amsterdam Leukodystrophy Center, Emma Children’s Hospital, Amsterdam UMC, Vrije Universiteit Amsterdam, Amsterdam, The Netherlands; 2 Molecular and Cellular Mechanisms, Amsterdam Neuroscience, Amsterdam, The Netherlands; 3 Department of Pathology, Amsterdam UMC, Vrije Universiteit Amsterdam, Amsterdam, The Netherlands; 4 Biomolecular Mass Spectrometry and Proteomics, Bijvoet Center for Biomolecular Research and Utrecht Institute for Pharmaceutical Sciences, University of Utrecht, Utrecht, The Netherlands; 5 Netherlands Proteomics Center, Utrecht, The Netherlands; 6 Department of Molecular Cell Biology and Immunology, Vrije Universiteit Amsterdam, Amsterdam, The Netherlands; 7 Department of Integrative Neurophysiology, Center for Neurogenomics and Cognitive Research, Vrije Universiteit Amsterdam, Amsterdam, The Netherlands

**Keywords:** Leukodystrophy, Quantitative proteomics, Disease progression, Brain, *2b5*^*ho*^ mouse model

## Abstract

Vanishing white matter (VWM) is a leukodystrophy caused by biallelic pathogenic variants in eukaryotic translation initiation factor 2B. To date, it remains unclear which factors contribute to VWM pathogenesis. Here, we investigated the basis of VWM pathogenesis using the *2b5*^*ho*^ mouse model. We first mapped the temporal proteome in the cerebellum, corpus callosum, cortex, and brainstem of *2b5*^*ho*^ and wild-type (WT) mice. Protein changes observed in *2b5*^*ho*^ mice were then cross-referenced with published proteomic datasets from VWM patient brain tissue to define alterations relevant to the human disease. By comparing *2b5*^*ho*^ mice with their region- and age-matched WT counterparts, we showed that the proteome in the cerebellum and cortex of *2b5*^*ho*^ mice was already dysregulated prior to pathology development, whereas proteome changes in the corpus callosum only occurred after pathology onset. Remarkably, protein changes in the brainstem were transient, indicating that a compensatory mechanism might occur in this region. Importantly, *2b5*^*ho*^ mouse brain proteome changes reflect features well-known in VWM. Comparison of the *2b5*^*ho*^ mouse and VWM patient brain proteomes revealed shared changes. These could represent changes that contribute to the disease or even drive its progression in patients. Taken together, we show that the *2b5*^*ho*^ mouse brain proteome is affected in a region- and time-dependent manner. We found that the *2b5*^*ho*^ mouse model partly replicates the human disease at the protein level, providing a resource to study aspects of VWM pathogenesis by highlighting alterations from early to late disease stages, and those that possibly drive disease progression.

**Supplementary Information:**

The online version contains supplementary material available at 10.1007/s00018-024-05258-4.

## Introduction

Vanishing white matter (VWM, OMIM #603,896) is a leukodystrophy that primarily manifests in young children [1]. It is clinically characterized by chronic neurological decline and stress-provoked episodes of rapid deterioration. These episodes are often followed by a partial or complete recovery, but may also lead to death [2–4]. The disease is caused by biallelic pathogenic variants in any of the 5 genes encoding the subunits of the eukaryotic translation initiation factor 2B (eIF2B) complex, which is a guanine exchange factor for eukaryotic translation factor 2 (eIF2) [5, 6]. As such, eIF2B is essential for regulating the initiation of translation and thus protein synthesis. eIF2B is in turn regulated through the phosphorylation of eIF2 during the integrated stress response (ISR). This is a control pathway activated in response to cellular stress [7, 8]. Because of the eIF2B defect, the ISR is constitutively dysregulated in VWM [9].

Defects in eIF2B cause neuropathology with degeneration of the white matter [2–4, 10]. Astrocytes are primarily affected, with secondary effects on oligodendrocytes and axons, explaining the lack of reactive gliosis, paucity of myelin, and axonal abnormalities in the white matter [3, 11–14]. The disease is more prominent in the white matter of telencephalic regions such as the frontal lobe, whereas other regions like the brainstem are often spared [3, 11]. Importantly, VWM also affects the gray matter, but to a lesser degree than the white matter [15]. Recent studies suggest that regional vulnerability in VWM may in part be caused by defects in proteins involved in cellular metabolism [15, 16]. It, however, remains unclear whether these molecular changes contribute to disease progression or if other factors are at play. Understanding the molecular pathogenesis of VWM is of importance in order to develop therapeutic strategies to abate, halt, or even reverse disease progression.

Mice with a homozygous point mutation in the *Eif2b5* gene (*Eif2b5*^*Arg191His/Arg191His*^, *2b5*^*ho*^) recapitulate VWM both clinically and pathologically [12]. Notably, this mutation is associated with severe disease in humans [17]. The *2b5*^*ho*^ mice are typically restricted in growth and have a clinical disease onset around 2–5 months of age, showing clear progressive gait ataxia. This is accompanied by myelin deficiency and vacuolization, axonal abnormalities, immature and dysmorphic astrocytes, immature oligodendrocytes, and mislocalized Bergmann glia, which are neuropathological hallmarks of human VWM [12]. On the basis of these clinical and pathological similarities, we assumed that the *2b5*^*ho*^ mouse model could be used to study molecular progression of VWM disease.

Here, we aimed at gaining more insight into the molecular basis of VWM pathogenesis. First, we investigated protein expression patterns in the *2b5*^*ho*^ mouse model using a data-independent mass spectrometry-based quantitative proteomic analysis. The proteome of 4 different brain regions was analyzed at different timepoints of disease progression. Brain regions were selected based on their regional vulnerability to VWM, and included the cerebellum, corpus callosum, cortex, and brainstem. We then assessed commonalities and differences in proteome changes between *2b5*^*ho*^ mouse and VWM patient brains to determine disease-relevant protein changes during disease development and progression.

## Materials and methods

### Animals

Experiments were performed using male mice with a homozygous point mutation in the *Eif2b5* (*Eif2b5*^*Arg191His/Arg191His*^, *2b5*^*ho*^) gene [12]. Wild-type (WT) C57BL/6J male mice were used as controls. Animals were weaned at P21 and kept at a 12 h light/dark cycle with ad libitum access to food and water. All experiments were reviewed and approved by the Animal Ethics Committee of the Central Authority for Scientific Procedures on Animals of the Netherlands (CCD) and the animal care and use committee of the Vrije Universiteit Amsterdam (protocol AVD1120020172804).

### Sample preparation for mass spectrometry analysis

Animals were sacrificed at the age of 1, 4, 7 and 12 months by cervical dislocation and brain tissue was removed for dissection. The cerebellum, corpus callosum, cortex, and brainstem were dissected. Dissected tissue samples were lysed, reduced and alkylated using the Sample Preparation by Easy Extraction and Digestion (SPEED) procedure [18]. First, samples were incubated in trifluoroacetic acid (TFA) in a 1:4 sample-to-TFA ratio for 20 min at room temperature, followed by neutralization with 2 M TrisBase in H_2_O. Protein samples were reduced and alkylated with 10 mM tris(2-carboxyethyl)phosphine (TCEP) and 40 mM chloroacetamide (CAA) for 5 min at 95 °C. Then samples were diluted 1:5 in H_2_O and digested at 37 °C using Lys-C (1:75 enzyme-to-substrate ratio) for 1 h followed by an overnight digestion with trypsin (1:50 enzyme-to-substrate ratio). Samples were acidified using formic acid (FA) to a final concentration of 4% (pH < 2), dried using a SpeedVac centrifuge, and desalted on the AssayMAP Bravo Platform with C18 cartridges (Agilent Technologies) following standard procedures. Briefly, C18 cartridges were primed and equilibrated with 80% acetonitrile (ACN)/0.1% FA and 0.1% FA, respectively. Peptide samples were resuspended in 0.1% FA, loaded on the cartridges and washed with 0.1% FA. Samples were eluted using 80% ACN/0.1% FA, dried using a SpeedVac centrifuge. To correct for systematic variations and batch effects, a pooled reference sample comprising all brain regions, timepoints and genotypes was prepared. Samples were stored at −20 °C until further use.

### Data-independent acquisition mass spectrometry analysis

Mass spectrometry (MS) data acquisition was performed on an Orbitrap Exploris 480 mass spectrometer connected to a Dionex Ultimate 3000 nano-UHPLC system (Thermo Fisher Scientific). For chromatographic separation of peptides, a micro-precolumn (C18 PepMap100, 5 µm, 100 Å, 5 mm × 300 μm; Thermo Fisher Scientific) coupled with an in-house produced analytical column (50 cm × 75 μm (ID) × 360 μm (OD), Reprosil-Pur 120 C18-AQ, 2.4 µm particle size) was used. Peptides were resuspended in 2% FA and approximately 0.4 µg peptides per sample were loaded in loading buffer solution (0.1% FA) at a flow rate of 30 µL/min. For the pooled reference sample, 0.2 µg peptides were injected. Samples were eluted with a linear gradient over 65 min from 13 to 44% solvent B (80% ACN, 0.1% FA) at a flow rate of 300 nL/min. The MS instrument was operated in a data-independent acquisition (DIA) mode applying mass windows of 20 *m*/*z* for peptide precursors isolation (1 *m*/*z* mass window overlap) covering sequentially the range of 400–1000 *m*/*z*. Isolated precursors were fragmented by higher-energy collisional dissociation fragmentation at normalized collision energy of 28%. Hybrid MS2 spectra of the DIA experiment were acquired in the Orbitrap mass analyzer at a resolution of 30,000. Additional MS1 full scans were recorded at a mass range of 375–1600 *m*/*z* and a resolution of 60,000. For both experiments (MS1 and DIA-MS2), instrument-defined optimal automatic gain control target settings (standard) and maximum injection times (auto) were used.

### DIA data processing

Recorded data files were processed and analyzed in library-free mode using DiaNN (v1.8.1) [19]. A spectral library was generated based on an in silico FASTA digest and making use of the deep learning prediction capabilities of DiaNN. Precursor ions were generated combining the SwissProt *Mus musculus* database (17,127 entries, release October 2022) and a contaminants database (MaxQuant, 245 entries, release April 2019) using the following parameters: protease specificity was set to trypsin/P, allowing up to 1 missed cleavage; fixed modification was set to carbamydomethylation on cysteine residues; and variable modification was set to N-terminal protein acetylation and oxidation of methionine residues with maximum variable modifications per peptide set to 1. Peptide lengths considered in the library ranged from 5 to 30 amino acids (precursor charges: 1–4) with a *m*/*z* range of 350–1650 *m*/*z* for precursor ions, and 100–2000 *m*/*z* for fragment ions. MS1 and MS2 mass accuracies as well as the scan window radius were determined automatically for each single experiment. Match-between-runs and heuristic protein inference was enabled, protein grouping/inference was performed based on the protein name. All other settings were left as default. Precursor false discovery rate (FDR) was set to 1% and all further generated precursor and protein quantifications were filtered with a Q cutoff value set to 1% on both the precursor and protein group level. Only proteotypic peptides and their respective proteins were considered for analysis. Further data processing was conducted using R statistical software (v1.3.1093). The pmp R package was employed to correct for signal drift and batch effects across runs [20]. For this, normalized precursor intensities for each entry in the DiaNN output report were extracted along with its precursor identifier and MS run. Missing values and non-reproducible peak intensities measured within quality control samples were filtered out with an 80% detection threshold. The remaining data were then corrected for signal drift and batch effects using the *QCRSC* algorithm with default settings. The corrected precursor intensity values were used for protein quantification applying the MaxLFQ algorithm with the DiaNN R package. Finally, the quantitative matrix output was stringently filtered to only contain proteins with valid values across all samples and samples with at least 1000 quantified values.

### Data analysis and visualization

Data were analyzed and visualized using R statistical software (v1.3.1093) and GraphPad prism (version 9.3.1). Venn diagrams were drawn using the online webtool provided by Bioinformatics & Evolutionary Genomics (http://bioinformatics.psb.ugent.be/webtools/Venn/). Spearman correlation analysis of intensity values of all biological samples was performed. Principal component analysis was conducted using FactoMineR R package [21]. Differential expressed proteins were identified using the limma R package [22]. Statistical significance was determined using the Benjamini–Hochberg’s (BH) adjustment for multiple comparison, defining an adjusted (adj.) *p* value of *p* < 0.05. Data were filtered to only include proteins with |log_2_ fold change (FC)| > 1. Gene ontology overrepresentation analysis for biological process and cellular component was performed using g:Profiler (version e109_eg56_p17_1d3191d, database updated on March 2023) [23]. For this, only driver terms with at least 2 annotated proteins were considered for analysis.

### Human VWM cerebellar white matter proteomics

An additional proteomics study was performed to analyze the proteome of the white matter in the cerebellum of 4 controls and 4 genetically proven VWM patients (Supplementary Table 1). Post mortem brain tissue from controls and VWM patients was collected at the Netherlands Brain Bank and the Amsterdam UMC location Vrije Universiteit Amsterdam, respectively. No confounding neuropathological or structural abnormalities were observed in the controls. Tissue was obtained within 6 h post mortem. Informed consent was obtained in all cases. The study was approved by the Medical Ethical Committee of the Amsterdam UMC location Vrije Universiteit Amsterdam and conducted according to the declaration of Helsinki. Fresh frozen tissue was processed for laser capture microdissection, mass spectrometry analysis, and differential protein expression analysis as described in our previous study [16].

### eIF2B activator proteome comparison

The *2b5*^*ho*^ mouse regional proteome profiles at different ages were compared to the cerebellar proteome profile of 7 month-old *2b5*^*ho*^ mice treated with an eIF2B activator. Proteomic data of the latter originate from a previously published study [24]. Only dysregulated proteins (|log_2_ FC| > 1, adj. *p* < 0.05) that were normalized by eIF2B activated were taken from this dataset and considered for further comparative analysis with the data generated in the present work.

### Human–mouse proteome comparison

Similarities and differences between protein changes in the brains of *2b5*^*ho*^ mice and VWM patients were assessed. Given the regional differences in disease severity, we compared mouse and human brain regions on the basis of their regional vulnerability in VWM: (1) *2b5*^*ho*^ mouse brainstem vs. VWM patient pons white matter, (2) *2b5*^*ho*^ mouse cortex vs. VWM patient cortex, (3) *2b5*^*ho*^ mouse cerebellum and corpus callosum vs. VWM patient cerebellar white matter, and (4) *2b5*^*ho*^ mouse cerebellum and corpus callosum vs. VWM patient frontal white matter. For this, we employed the VWM patient cerebellar white matter proteome data obtained as described above, and 2 recently reported proteome datasets of the cortex (gray matter) and the white matter of the middle frontal gyrus and the pons of VWM patients (PXD030831 and PXD040861) [15, 16]. First, human orthologues were retrieved for proteins differentially expressed (|log_2_ FC| > 1, adj. *p* < 0.05) in each individual brain regions of 12 months old *2b5*^*ho*^ mice using the g:Profiler web tool [23]. Then overlap in protein changes between mouse and human was determined. Only overlapping proteins that were concordantly dysregulated in both mouse and human were considered for analysis.

### Immunohistochemistry

Mouse and human brain tissue sections were stained according to standard protocols using the antibodies listed in Table 1. Briefly, 5 µm thick formalin-fixed, paraffin-embedded brain tissue was deparaffinized and incubated in 0.3% H_2_O_2_ in H_2_O for 30 min. Then heat-induced antigen retrieval was performed in 10 mM citrate buffer (pH 6.0) or Tris/EDTA buffer (pH 9.0). Immunopositivity was visualized using 3,3′-diaminobenzidine (DAB) chromogen, and hematoxylin was used as counterstain. Images were acquired using a Leica DM4000B light microscope (Leica Microsystems) using a 200× objective.

**Table 1:** List of antibodies used for immunohistochemistry

Antibody	Dilution	Vendor	Catalog #
SLC7A5	1:200	Abcam	ab208776
TNC	1:200	Abcam	ab108930
PYGM	1:2000	ThermoFisher	MA5-27442
BCAN	1:100	ThermoFisher	PA5-52477
GFAP	1:750	Merck Millipore	AB5541

## Results

### Mapping spatiotemporal brain proteome in the *2b5*^*ho*^ mouse

**Fig. 1:**
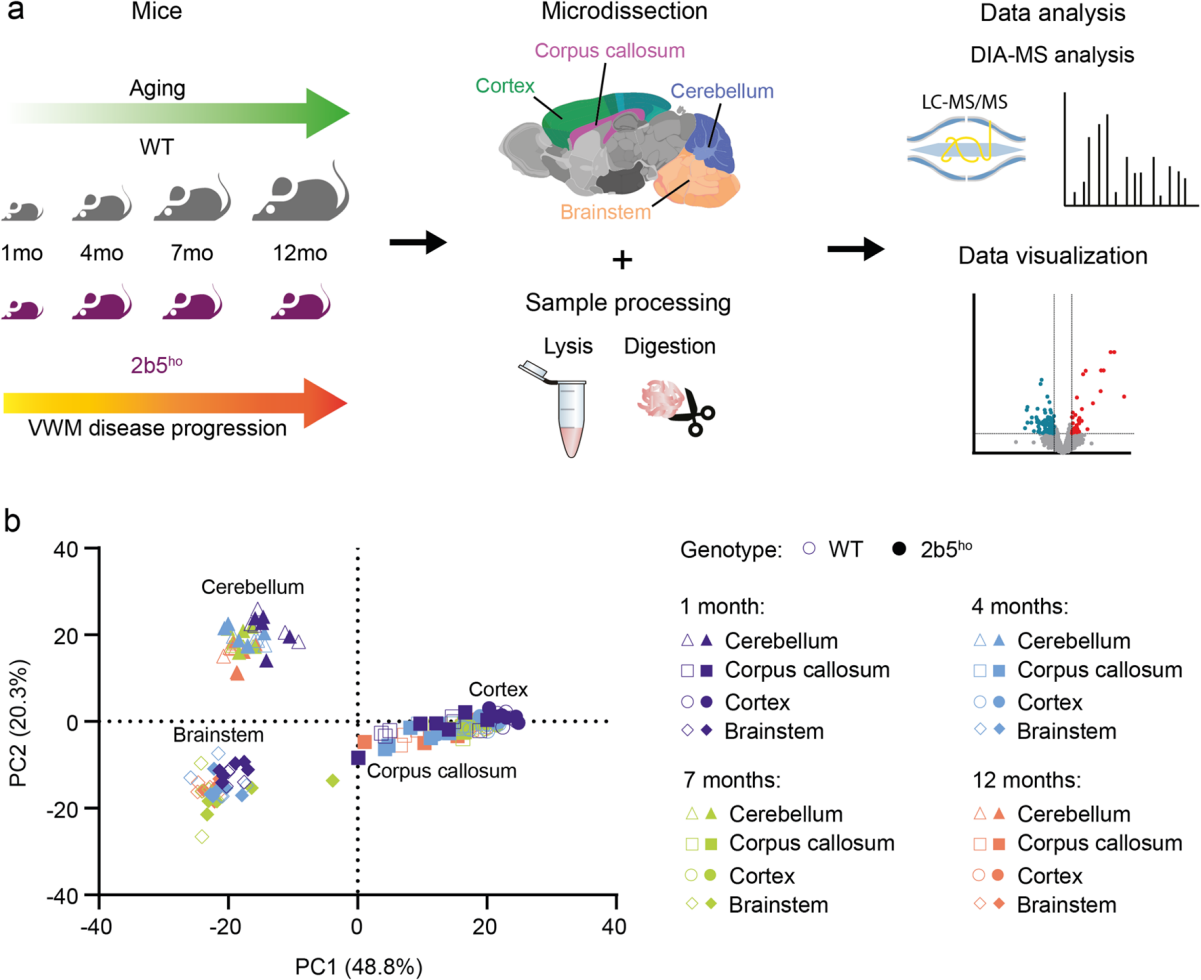
Mapping spatiotemporal brain proteome in WT and *2b5*^*ho*^ mouse. **a** Experimental design. Protein expression profiles in selected brain regions of WT and *2b5*^*ho*^ mice at 1, 4, 7 and 12 months of age are mapped by high-resolution mass spectrometry-based proteomics. Mass spectrometry data are acquired using a DIA approach and further subjected to downstream bioinformatics analysis for data analysis and visualization. **b** Principal component analysis of the WT and *2b5*^*ho*^ mouse brain proteomes shows clustering of samples based on brain region. The first component (PC1), which explains 48.8% of the variability, separates the cerebellum and brainstem from the corpus callosum and cortex. The second component (PC2) highlights segregation between samples from the cerebellum and brainstem, accounting for 20.3% of the variability

To gain insight into the molecular disease progression in VWM, we mapped protein expression changes along the spatiotemporal axis in WT and *2b5*^*ho*^ mouse brains using high-resolution mass spectrometry-based proteomics. For this, we selected 4 different timepoints, representing different stages in the disease. These included an early timepoint before clinical symptom onset at 1 month (pre-symptomatic); a timepoint at clinical symptom onset at 4 months (early-symptomatic); a late timepoint at 7 months (symptomatic); and a humane end point at 12 months at the end of the lifespan of the *2b5*^*ho*^ mice (disease end-stage). We microdissected the brain into 4 different regions (i.e. cerebellum, corpus callosum, cortex, and brainstem), which are differently affected in *2b5*^*ho*^ mice, with cerebellum and corpus callosum representing more severely affected regions and brainstem and cortex being relatively spared (Fig. 1a).

Proteome analysis identified a total of 5548 proteins, and only a subset of 3706 proteins with quantitative values in all samples were considered for further analysis (Supplementary Table 2). Correlation analysis revealed a moderate correlation between all biological samples (Pearson correlation ranging from 0.5979 to 0.9989). Biological samples within brain regions showed a high degree of reproducibility between each other (Supplementary Fig. 1). Notably, we observed a strong correlation between biological samples from the corpus callosum and cortex at each timepoint (Pearson correlation ranging from 0.8915 to 0.9979), indicating comparable protein expression patterns (Supplementary Fig. 1). Principal component analysis showed a clear separation of samples based on brain region with clustering of samples from the corpus callosum and cortex (Fig. 1b).

### Spatiotemporal proteome changes in the *2b5*^*ho*^ mouse brain

**Fig. 2:**
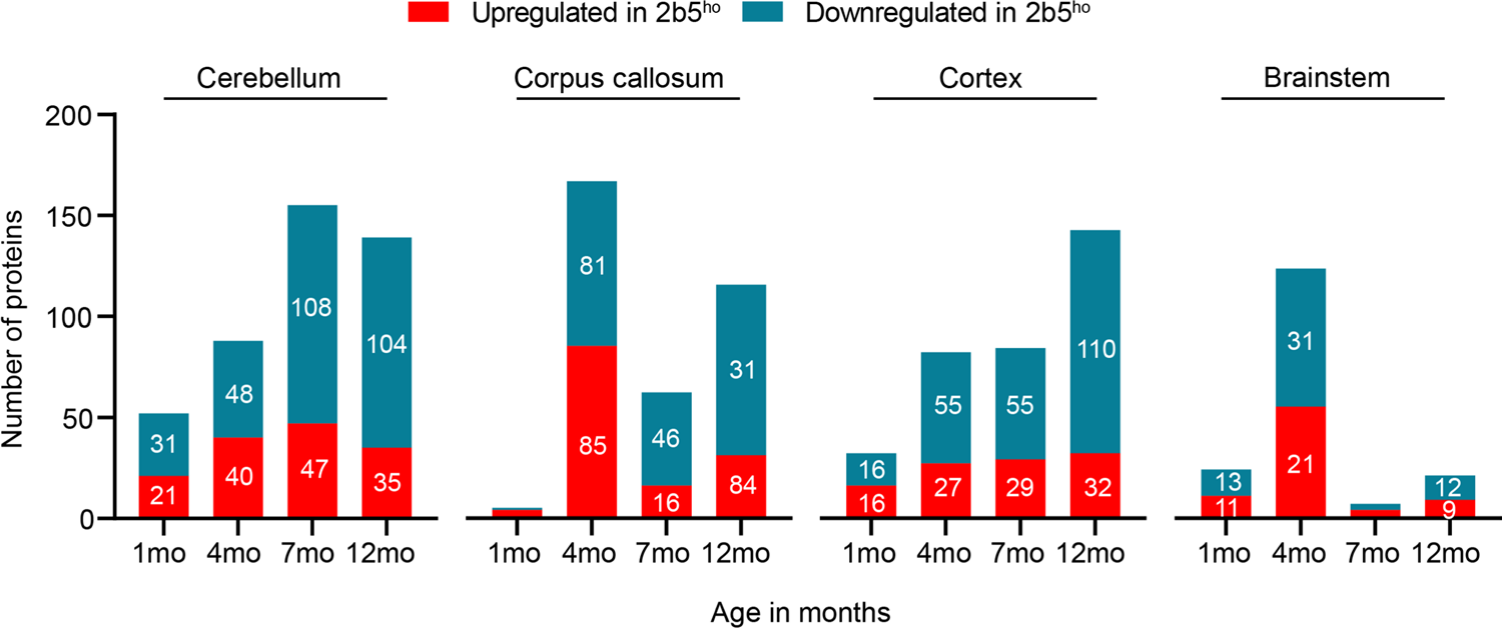
Spatiotemporal protein expression changes in the *2b5*^*ho*^ mouse brain. Number of significantly dysregulated proteins (|log_2_ FC| > 1, adj. *p* < 0.05) in the cerebellum, corpus callosum, cortex, and brainstem of *2b5*^*ho*^ compared to WT mice at different ages. Bars represent the number of significantly downregulated (blue) and upregulated (red) proteins

We first analyzed global protein expression changes by comparing individual brain regions of *2b5*^*ho*^ mice at different ages with their corresponding WT counterparts (Supplementary Fig. 2; Supplementary Table 3). Subtle proteome changes were already observed at 1 month (pre-symptomatic) in the cerebellum, cortex, and brainstem, while the corpus callosum remained relatively unaffected (Fig. 2). Three months later at clinical symptom onset, substantial protein changes were detected in all brain regions. Amongst the 4 brain regions, the corpus callosum showed the strongest proteome response at this timepoint, with a total of 166 differentially expressed proteins (Fig. 2). Notably, protein changes increased in the cerebellum and cortex as the disease progressed. By contrast, a decrease in protein changes was observed in the corpus callosum from 7 months onwards. Remarkably, the proteome in the brainstem appeared relatively unaffected from 7 months onwards (Fig. 2). So far, our findings suggest that protein expression profiles of the examined brain regions in the *2b5*^*ho*^ mice are differently affected. More importantly, protein changes occur highly time-dependent, with the number of protein changes increasing or decreasing in a region-dependent manner as the disease progressed.

**Fig. 3:**
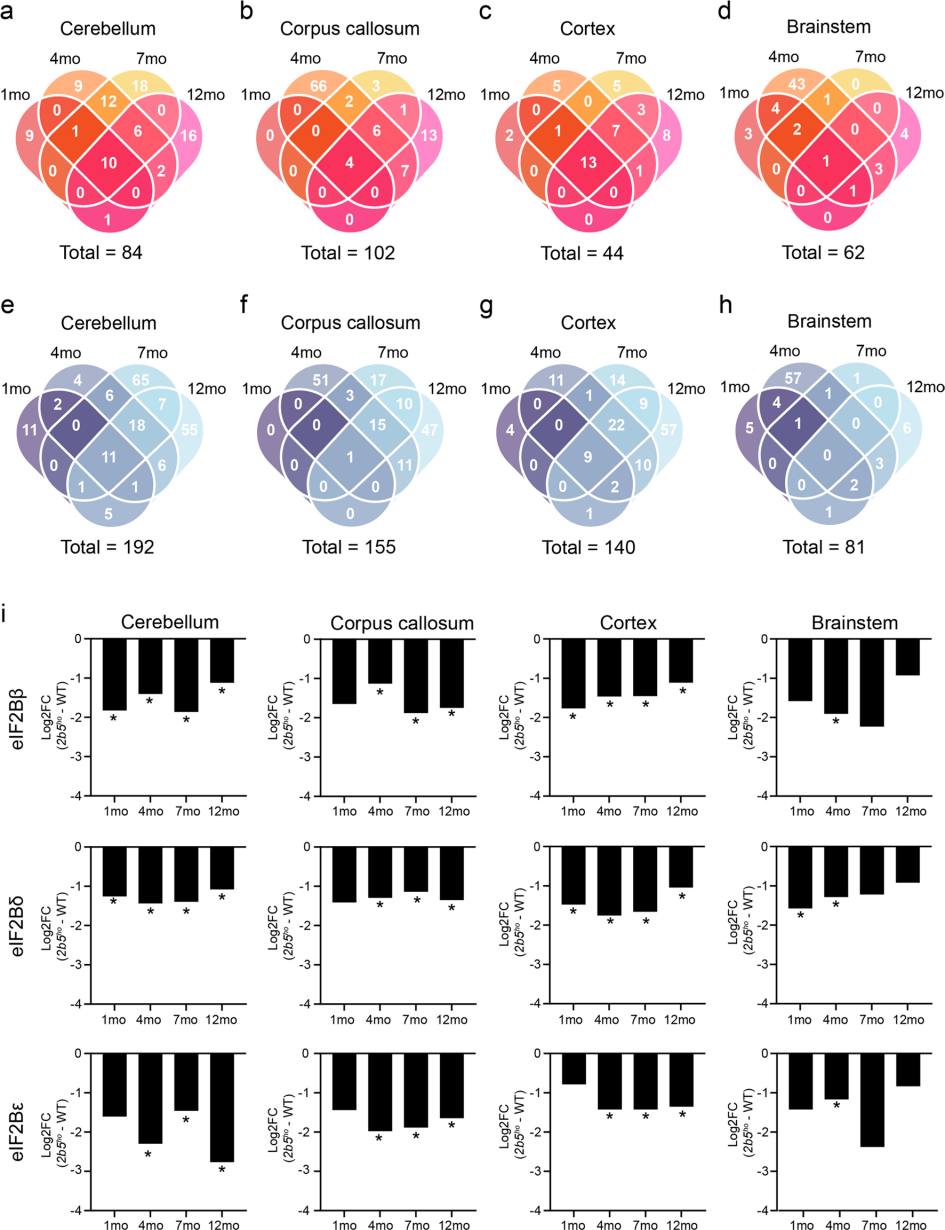
Proteome changes throughout the disease course in the *2b5*^*ho*^ mouse brain. Venn diagrams showing number of protein changes consistently **a**–**d** upregulated or **e**–**h** downregulated over time in the cerebellum, corpus callosum, cortex, and brainstem. **i** Temporal expression changes of proteins associated with the subunits of the eIF2B complex in the *2b5*^*ho*^ mouse brain. Data are presented as log_2_ FC (*2b5*^*ho*^ vs. WT) over time. * *p* < 0.05

We next investigated protein changes associated with disease progression. Analysis revealed that the cerebellum, corpus callosum, cortex, and brainstem of *2b5*^*ho*^ mice exhibited a total of 276, 257, 184, and 143 protein changes (|log_2_ FC| > 1, adj. *p* < 0.05), respectively, that occur at least at one timepoint (Fig. 3a–h). The regional differences in protein changes possibly reflect differential vulnerability of the brain regions to the defect in eIF2B. We decided to specifically focus on differentially expressed proteins that displayed a consistent change—either up- or downregulated—until the end of the *2b5*^*ho*^ mice lifespan. A gene ontology overrepresentation analysis was conducted to determine alterations in biological functions occurring over time. Results of the gene ontology overrepresentation analysis are listed in Supplementary Table 4.

**Fig. 4:**
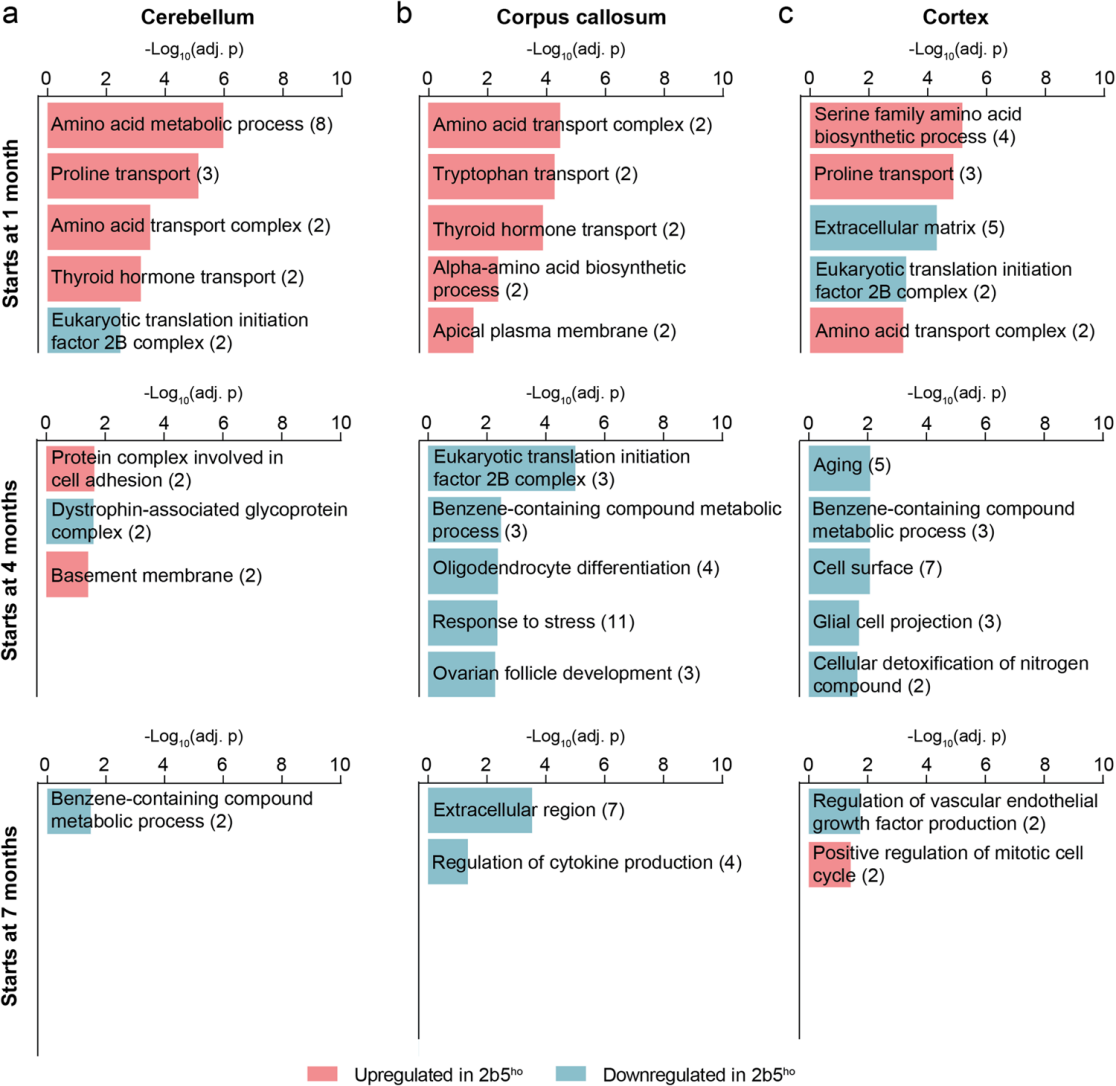
Spatiotemporal changes in biological function in the *2b5*^*ho*^ mouse brain. Gene ontology overrepresentation analysis of proteins with consistent alterations in expression level starting at 1, 4 or 7 months of age in the **a** cerebellum **b** corpus callosum, and **c** cortex of *2b5*^*ho*^ mice. Graphs showing the top 5 overrepresented gene ontology term based on −log_10_(adj. *p*) value. Bars represent ontology terms associated with downregulated (blue) and upregulated (red) proteins. The number of proteins functionally annotated to each term are shown between brackets

In the cerebellum, we found 21 proteins significantly changed starting at 1 month, with 10 and 11 proteins being consistently up- and downregulated, respectively (Fig. 3a, e). Analysis revealed that many upregulated proteins were involved in amino acid metabolic process (ALDH18A1, CTH, ASNS, PHGDH, PSAT1, EPRS1, CARS1) (Fig. 4a; Supplementary Table 4). Some of the downregulated proteins contained those associated with the eIF2B complex (eIF2Bβ, eIF2Bδ). Notably, eIF2Bε was found downregulated in the cerebellum starting at 4 months. Further analysis revealed that 24 and 7 additional proteins started to consistently change in expression levels from 4 to 7 months onwards, respectively (Fig. 3a, e). Remarkably, only a few of these proteins were functionally annotated (Fig. 4a; Supplementary Table 4).

In the corpus callosum, only 5 proteins showed consistent alteration in expression levels starting at 1 month (Fig. 3b, f). This is of no surprise since little proteome changes were detected at 1 month before clinical symptom onset (Fig. 2). Amongst the 5 proteins, some were involved in amino acid transport (SLC7A5, SLC3A2) (Fig. 4b; Supplementary Table 4). At 4 months, 21 additional proteins were identified with consistent changes in expression level over time (Fig. 3b, f). Of these, 6 proteins were consistently upregulated, of which some were associated with response to cytokine (SHMT2, EPRS1, CTH, PCK2) (Supplementary Table 4). The remaining 15 proteins were consistently reduced in expression levels. Interestingly, some of these proteins were involved in response to stress (GSTM1, CST3, EPHX2, GSTM5, APOE, ENTPD2, eIF2Bδ, VCAM1, eIF2Bε, eIF2Bβ, PLPP3) and oligodendrocyte differentiation (DAAM2, eIF2Bδ, eIF2Bε, eIF2Bβ) (Fig. 4b; Supplementary Table 4). Notably, proteins of the eIF2B complex were also downregulated at this timepoint. At 7 months, 11 proteins started to consistently alter in expression level (Fig. 3b, f). Many of the downregulated proteins were associated with the extracellular region (S100A13, BCAN, AQP4, BTBD17, HPX, S100A16, GPRC5B) or involved in the regulation of cytokine production (TRIB2, S100A13, AQP4, GPRC5B) (Fig. 4b; Supplementary Table 4).

In the cortex, 22 proteins were consistently altered starting at 1 month (Fig. 3c, g). Of these, 13 proteins consistently increased in expression over time with some being involved in amino acid biosynthetic process (PSPH, PSAT1, CTH, SHMT2) (Fig. 4c; Supplementary Table 4). The remaining 9 proteins were consistently decreased in expression over time. Some of these proteins were associated with the extracellular matrix (APOE, CLU, CST3, S100A13, SPARCl1) and eIF2B complex (eIF2Bβ, eIF2Bδ), or involved in oligodendrocyte differentiation (CLU, eIF2Bβ, eIF2Bδ, DAAM2) (Fig. 4c; Supplementary Table 4). Starting at 4 and 7 months, 29 and 12 additional proteins with consistent alterations over time were identified, respectively (Fig. 3c, g). Only a small proportion of these proteins, however, were functionally annotated (Fig. 4c; Supplementary Table 4).

Remarkably, in the brainstem only 1 protein (ALDH18A1) increased in expression over time starting at 1 month of age (Fig. 3d, h). No additional proteins with consistent changes in expression level at later disease stages were identified (Fig. 3d, h). This is consistent with the little proteome changes found in the brainstem after 4 months (Fig. 2), suggesting that proteome changes in the brainstem are transient following symptom onset.

Overall, our data suggest that disease gradually progresses across all regions except the brainstem. Furthermore, spatiotemporal protein changes reflect alterations in several biological functions. Notably, proteins of the eIF2B complex were affected in the individual brain regions at different timepoints (Fig. 3i). These protein changes are likely caused by the defect in eIF2B. In the cerebellum and cortex, protein changes associated with the eIF2B complex already started before clinical symptom onset, whereas in the corpus callosum they only occurred later at symptom onset (4 months). In the brainstem, these protein changes also appeared starting at 4 months, but were transient and not observed later in the disease process (Supplementary Table 3). Notably, these time-dependent changes in eIF2B complex-related proteins partially coincided with the changes in spatiotemporal protein expression patterns observed in the *2b5*^*ho*^ mouse brain (Fig. 2).

### Protein changes in the *2b5*^*ho*^ mouse brain display a monotonic or non-monotonic behavior over time

**Fig. 5:**
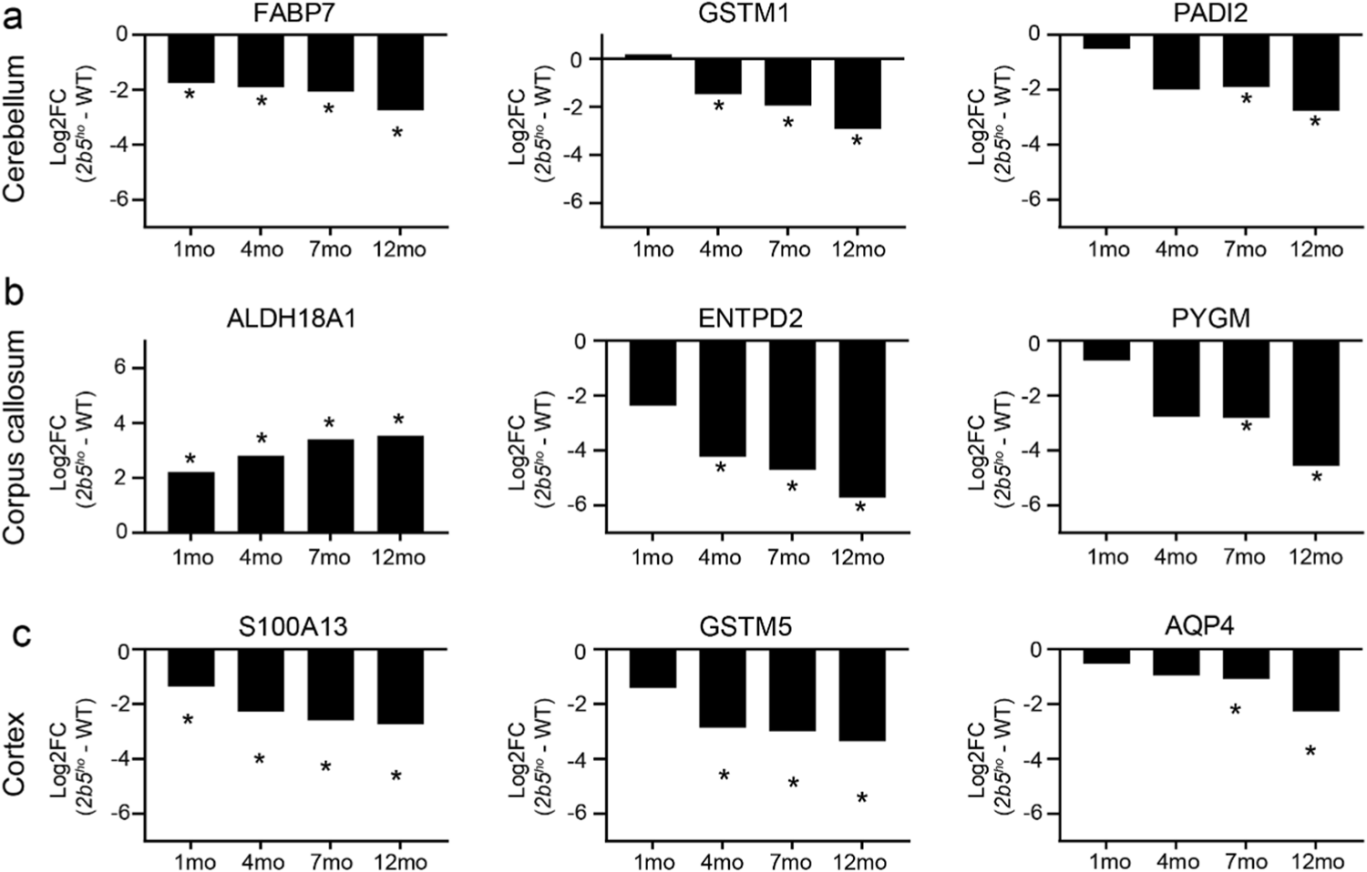
Dysregulated proteins in the *2b5*^*ho*^ mouse brain showing monotonic behavior. A selection of dysregulated proteins displaying a monotonic behavior starting at different timepoints in the **a** cerebellum, **b** corpus callosum, and **c** cortex. Data are presented as log_2_ FC (*2b5*^*ho*^ vs. WT) over time. * *p* < 0.05

We next investigated whether proteins with consistent up- or downregulation in expression level over time displayed a monotonic behavior. Proteins showing a monotonic behavior are those with continuous increase or decrease in expression levels as the disease progresses. These could contribute as molecular drivers of pathology in the *2b5*^*ho*^ mouse brain, but could also reflect increasing damage. We found a total of 11, 14, and 16 proteins that displayed a monotonic behavior in the cerebellum, corpus callosum, and cortex, respectively (Table 2). A selected number of proteins displaying a monotonic behavior starting at different time points are shown in Fig. 5.

**Table 2:** List of differentially expressed proteins displaying monotonic behavior over time in the cerebellum, corpus callosum and cortex of *2b5*^*ho*^ mice

Gene	1 month		4 months		7 months		12 months		Direction
	Log_2_ FC	Sig	Log_2_ FC	Sig	Log_2_ FC	Sig	Log_2_ FC	Sig	
*Cerebellum*									
FABP7	−1.7448	*	−1.9160	*	−2.0658	*	−2.7420	*	−
FARP1	−0.7238		−1.1153	*	−1.1940	*	−1.3232	*	−
GSTM1	0.1812		−1.4666	*	−1.9419	*	−2.9096	*	−
DMD	−0.9927		−1.0861	*	−1.0904	*	−1.6756	*	−
TST	−0.9703		−1.9459	*	−2.1322	*	−2.8509	*	−
SLC20A2	−1.3928		−1.9336	*	−2.0998	*	−2.4028	*	−
PADI2	−0.5073		−1.9925		−1.8960	*	−2.7702	*	−
GSTM5	−0.7849		−1.4508		−1.4684	*	−2.6646	*	−
FAH	−0.8680		−1.3036		−1.2113	*	−1.9931	*	−
PHKA1	0.2119		−0.7305		−1.0456	*	−1.0974	*	−
PACSIN3	−0.0573		−1.0630		−1.2164	*	−1.3515	*	−
*Corpus callosum*									
ALDH18A1	2.2159	*	2.8065	*	3.4106	*	3.5381	*	+
APOE	−2.4899		−2.8878	*	−3.5316	*	−3.8432	*	−
ENTPD2	−2.3678		−4.2262	*	−4.6981	*	−5.7154	*	−
PADI2	−0.8893		−1.4105	*	−2.4699	*	−2.7876	*	−
PLPP3	−0.8168		−1.9522	*	−1.9655	*	−2.4991	*	−
PRDX6	−0.6511		−1.4579	*	−1.7522	*	−2.0964	*	−
TRIB2	−0.5184		−0.8916		−1.7999	*	−2.3802	*	−
PYGM	−0.7210		−2.7753		−2.8136	*	−4.5587	*	−
SNTA1	−0.2522		−0.9914		−1.3034	*	−1.5271	*	−
BCAN	−0.3069		−0.7949		−1.3836	*	−1.4765	*	−
AQP4	−0.3106		−0.9279		−2.4731	*	−2.9274	*	−
BTBD17	−0.4788		−1.4798		−1.3911	*	−2.9514	*	−
HPX	−1.1550		−2.1227		−2.1382	*	−5.8252	*	−
S100A16	−0.7662		−0.7553		−1.3219	*	−1.5962	*	−
*Cortex*									
ALDH18A1	2.2115	*	2.87131	*	3.2593	*	3.3322	*	+
S100A13	−1.3615	*	−2.2779	*	−2.5919	*	−2.7313	*	−
VCAM1	−0.2709		−1.7120	*	−1.7715	*	−2.2071	*	−
PYGM	−0.3689		−2.7596	*	−2.9901	*	−3.5778	*	−
GSTM5	−1.4126		−2.8634	*	−2.9876	*	−3.3506	*	−
GSTM1	−0.6922		−2.6524	*	−3.0784	*	−3.2481	*	−
TRIB2	−0.4018		−1.2728	*	−1.3182	*	−1.7776	*	−
BCAN	−0.6661		−1.0400	*	−1.2805	*	−1.4039	*	−
RCC2	0.7156		0.8626		1.0781	*	1.2743	*	+
CSRP1	−0.5508		−0.9802		−1.0446	*	−1.2964	*	−
PADI2	−1.1363		−1.3306		−1.8857	*	−2.4950	*	−
CBR3	−0.5545		−0.9814		−1.1772	*	−1.4007	*	−
SLC25A18	−0.3088		−1.1779		−1.6799	*	−1.7634	*	−
C1ORF198	−0.8104		−0.7930		−1.4547	*	−2.0346	*	−
NDRG2	−0.5820		−1.0439		−1.3447	*	−1.6239	*	−
AQP4	−0.5212		−0.9520		−1.0812	*	−2.2684	*	−

+/− indicates directional change of protein expressions

* Differentially expressed proteins (|log_2_ FC| > 1, adj. *p* < 0.05)

We observed that the number of proteins that follow a monotonic trend increased over time in these regions. Notably, the majority of these proteins in each individual brain region continuously decreased in expression over time. By contrast, we did not observe any proteins that continuously increased or decreased in the brainstem as the disease progressed.

### The ISR is affected over time in the *2b5*^*ho*^ mouse brain at the protein level

**Fig. 6:**
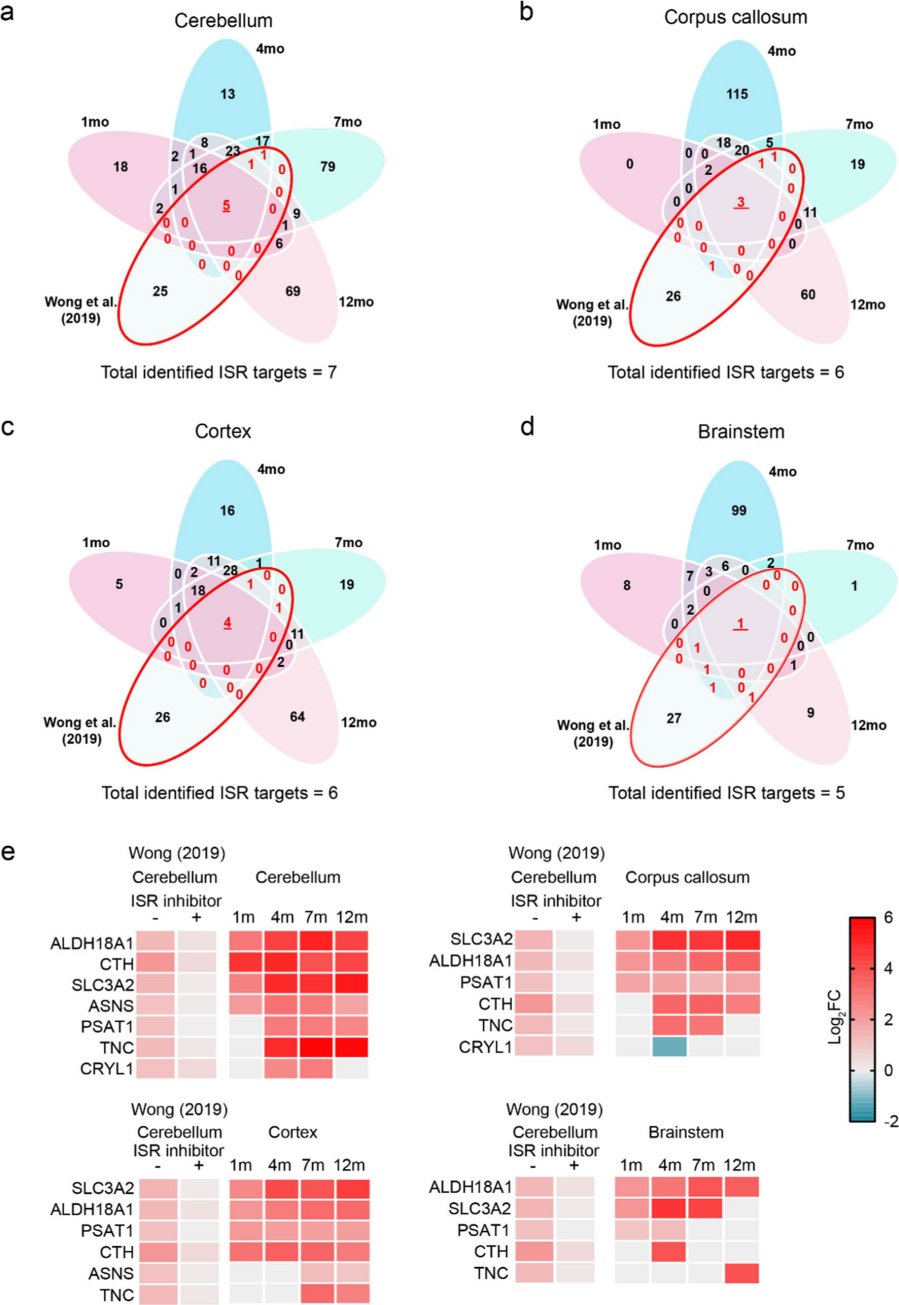
Comparison of dysregulated proteins with an independent cerebellum proteomic dataset of 7-month-old *2b5*^*ho*^ mice treated with ISR inhibitor. **a**–**d** Venn diagrams showing the overlap of regional dysregulated proteins identified in *2b5*^*ho*^ mice of different ages (present work) and a previously published proteomic study [24]. Only dysregulated proteins that were normalized in expression by ISR inhibitor treatment were considered for comparison to the present work. The number of ISR targets identified in at least 1 timepoint or all timepoints are shown in red. **e** Heatmaps showing expression patterns of overlapping dysregulated ISR protein targets found between the two proteomic datasets

Since the ISR is constitutively dysregulated in VWM [9], we next investigated how it is affected in individual brain regions of *2b5*^*ho*^ mice throughout the disease course. For this, we employed a previously published proteomic study, in which the cerebellum of 7-month-old *2b5*^*ho*^ mice carrying the same pathogenic variant and treated with an ISR inhibitor was analyzed [24]. In this study, a total of 33 dysregulated proteins (|log_2_ FC| > 1, adj. *p* < 0.05) were found in the cerebellum of *2b5*^*ho*^ mice. Amongst these dysregulated proteins, most were normalized by ISR inhibitor treatment, except for one. Comparison of this dataset with our spatiotemporal proteomic data could reveal changes in ISR targets at the protein level in differently affected brain regions of the *2b5*^*ho*^ mouse brain over time. In our proteomic dataset, we identified a total of 7, 6, 6, and 5 ISR protein targets (|log_2_ FC| > 1, adj. *p* < 0.05) in the cerebellum, corpus callosum, cortex, and brainstem of *2b5*^*ho*^ mice, respectively (Fig. 6a–d). Further analysis revealed that changes in protein expression of the identified ISR-related targets occur at least at one time point across the *2b5*^*ho*^ mouse brain (Fig. 6e). Amongst these, only a few were affected from before clinical disease onset (1 month of age) until the end of the *2b5*^*ho*^ mice lifespan and include known ISR targets such as ASNS, CTH, SLC3A2 and PSAT1 (Fig. 6e).

### Dysregulated proteins in the *2b5*^*ho*^ mouse brain show considerable overlap with proteome changes detected in post mortem brain tissue of VWM patients

**Fig. 7:**
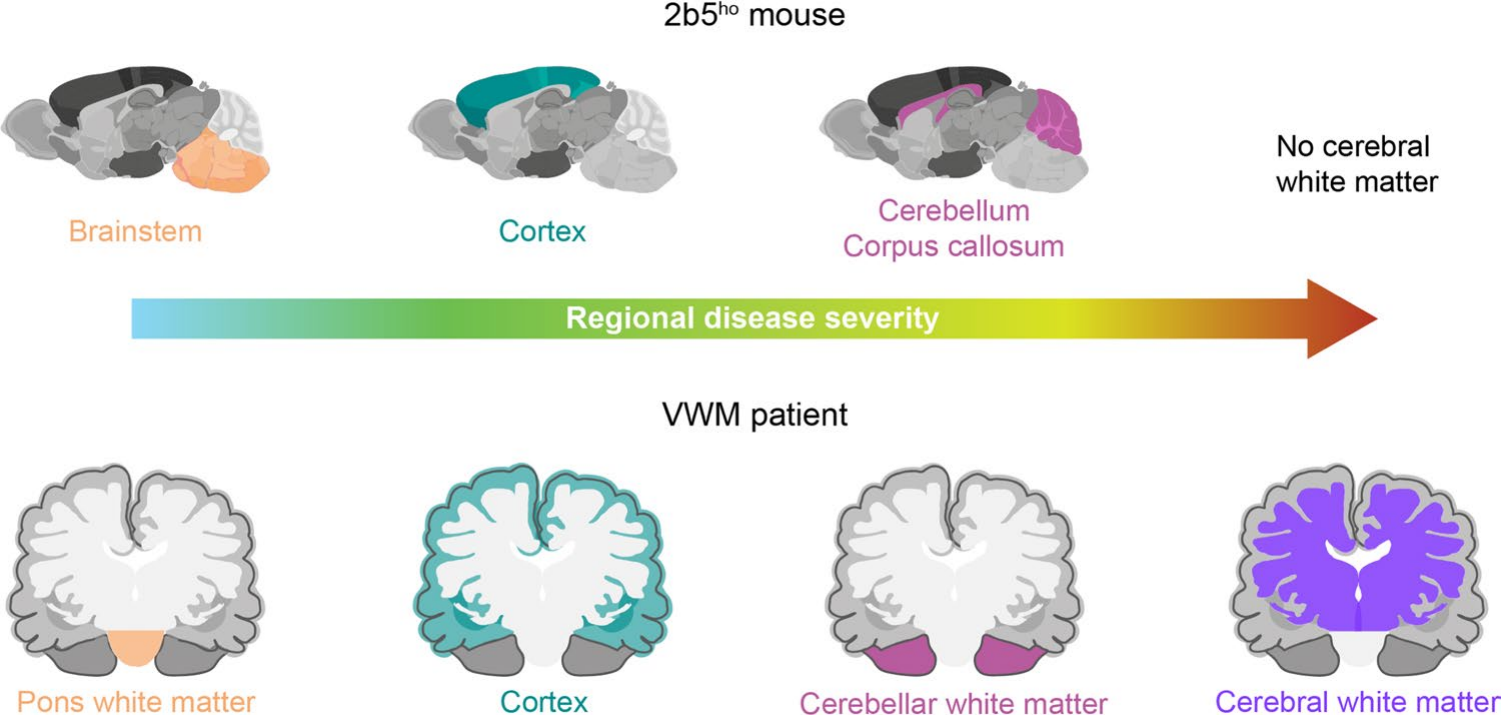
Brain regional vulnerability in pathology in *2b5*^*ho*^ mice and VWM patients. In *2b5*^*ho*^ mice, the brainstem and cortex appear relatively spared. Pathology in the *2b5*^*ho*^ brainstem and cortex are comparable with the pons white matter and cortex of VWM patients, respectively. The cerebellum and corpus callosum in the *2b5*^*ho*^ mouse brain share similar pathology with the cerebellar white matter in VWM patients. These regions are affected, but show no signs of cystic degeneration. The cerebral white matter is the most severely affected region in patients. No cerebral white matter is present in the mouse brain

Having established the spatiotemporal proteome in WT and *2b5*^*ho*^ mouse brain, we next asked to which extent *2b5*^*ho*^ mice recapitulate VWM disease in humans. A human to mouse comparison could reveal patient- and disease-relevant protein changes. For this, we employed proteomic data of the cortex (gray matter) and the white matter of the middle frontal gyrus, cerebellum and pons of VWM patients [15, 16]. In these datasets, a total of 373, 474, 34, and 223 significantly differentially expressed proteins (|log_2_ FC| > 1, adj. *p* < 0.05) were identified in the cortex, frontal white matter, cerebellar white matter, and pons white matter of VWM patients, respectively [15, 16]. Given the regional differences in disease severity, we compared mouse and human brain regions on the basis of their regional vulnerability in VWM (Fig. 7). Changes observed in human post mortem tissue reflect the end-stage of the disease. Because of this, we first focused on proteins displaying changes in expression level at the end of the *2b5*^*ho*^ mice lifespan, and assessed whether these overlapped with proteome changes detected in post mortem brain tissue of VWM patients. Only overlapping proteins that are concordantly up- or downregulated in both mouse and human were considered for further analysis (Supplementary Table 5).

**Fig. 8:**
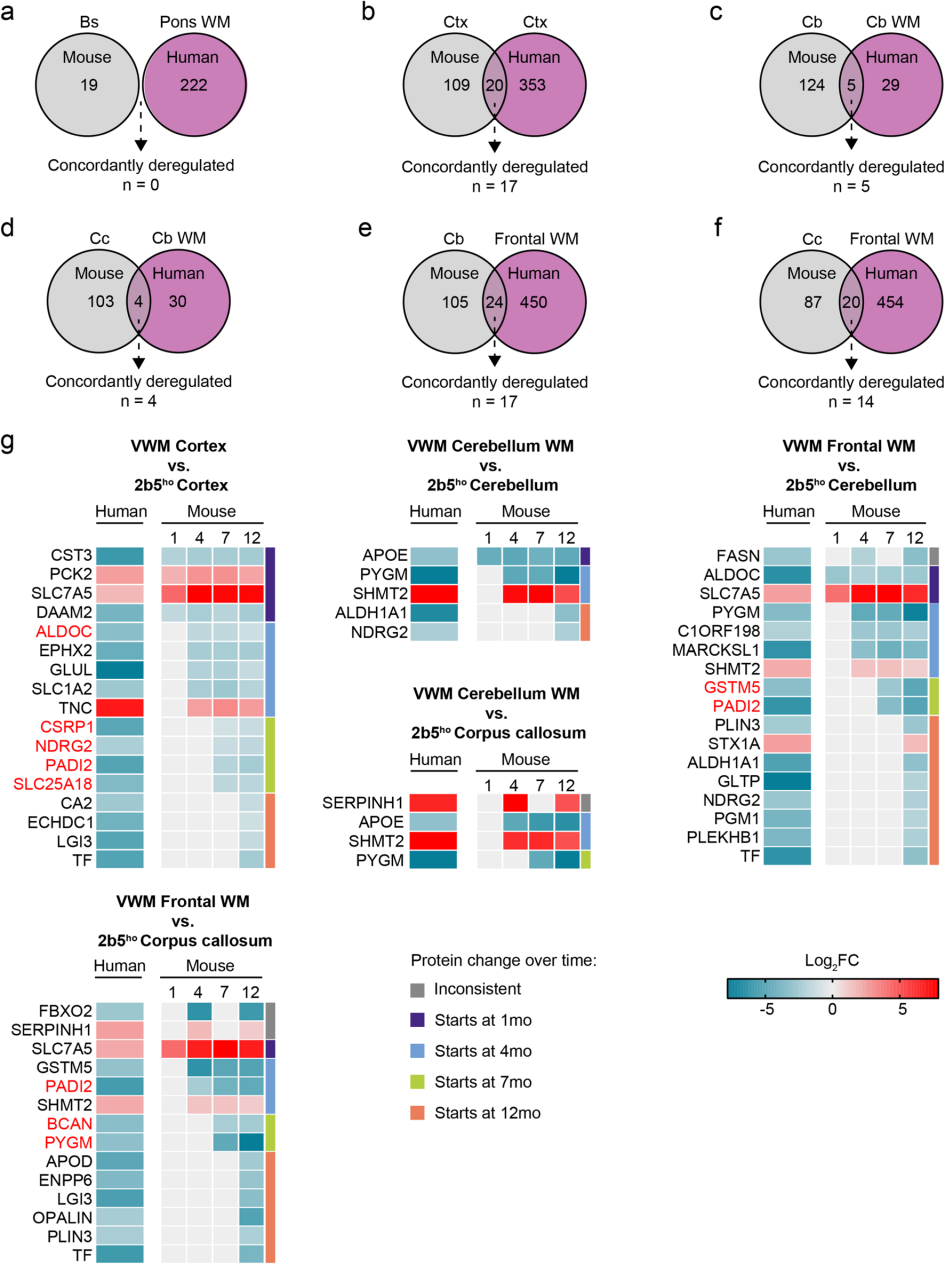
Comparison of brain proteome changes in *2b5*^*ho*^ mice and VWM patients.** a**–**f** Venn diagrams showing overlap in protein changes between *2b5*^*ho*^ mice (grey circle) and VWM patients (purple circle). The number of proteins concordantly dysregulated in both mouse and human is noted. Mouse and human brain regions are compared on the basis of their regional vulnerability in the disease: **a** *2b5*^*ho*^ brainstem (bs) vs. patient pons white matter (Pons WM), **b** *2b5*^*ho*^ cortex (ctx) vs. patient cortex (ctx), **c** *2b5*^*ho*^ cerebellum (Cb) vs. patient cerebellar white matter (Cb WM), **d** *2b5*^*ho*^ corpus callosum (Cc) vs. patient cerebellar white matter (Cb WM), **e** *2b5*^*ho*^ cerebellum (Cb) vs. patient frontal white matter (Frontal WM), and **f** *2b5*^*ho*^ corpus callosum (Cc) vs. patient frontal white matter (Frontal WM). **g** Heatmaps showing expression patterns of proteins that are concordantly up- or downregulated in both *2b5*^*ho*^ mice and VWM patients over time. Colored bars indicate proteins with consistent changes in expression level starting at 1 (purple), 4 (blue), 7 (green), or 12 (orange) months of age. Grey bar indicates proteins with inconsistent changes in expression level over time. Proteins displaying a monotonic behavior are highlighted in red

We assessed commonalities in proteome changes detected in the relatively preserved brain regions in *2b5*^*ho*^ mice and VWM patients (Fig. 7). Remarkably, no similarities were observed between the proteome of the brainstem in 12-month-old *2b5*^*ho*^ mice and the pons white matter in VWM patients (Fig. 8a; Supplementary Table 5). In the cortex of 12-month-old *2b5*^*ho*^ mice, 17 proteins were concordantly dysregulated in VWM patient cortex (Fig. 8b). We next examined the affected regions in *2b5*^*ho*^ mouse and VWM patient brains. For this, protein changes in the cerebellum and corpus callosum of 12-month-old *2b5*^*ho*^ mice were compared to those detected in the cerebellar white matter of VWM patients. Analysis revealed that only 5 and 4 proteins in the *2b5*^*ho*^ cerebellum and corpus callosum, respectively, were concordantly altered in the VWM patient cerebellar white matter (Fig. 8c, d; Supplementary Table 5). Since there is a lack of cerebral white matter in the mouse brain, we also compared *2b5*^*ho*^ cerebellum and corpus callosum with the frontal white matter of VWM patients (Fig. 8e, f; Supplementary Table 5). Comparison revealed a total of 17 and 14 proteins in the *2b5*^*ho*^ cerebellum and corpus callosum, respectively, concordantly altered in the VWM patient frontal white matter. This suggests that the *2b5*^*ho*^ mouse cerebellum and corpus callosum are more similar to the frontal than the cerebellar white matter of VWM patients at the protein level. Further examination of the concordantly altered proteins revealed that their expression levels changed time-dependently (Fig. 8g; Supplementary Table 5).

**Fig. 9:**
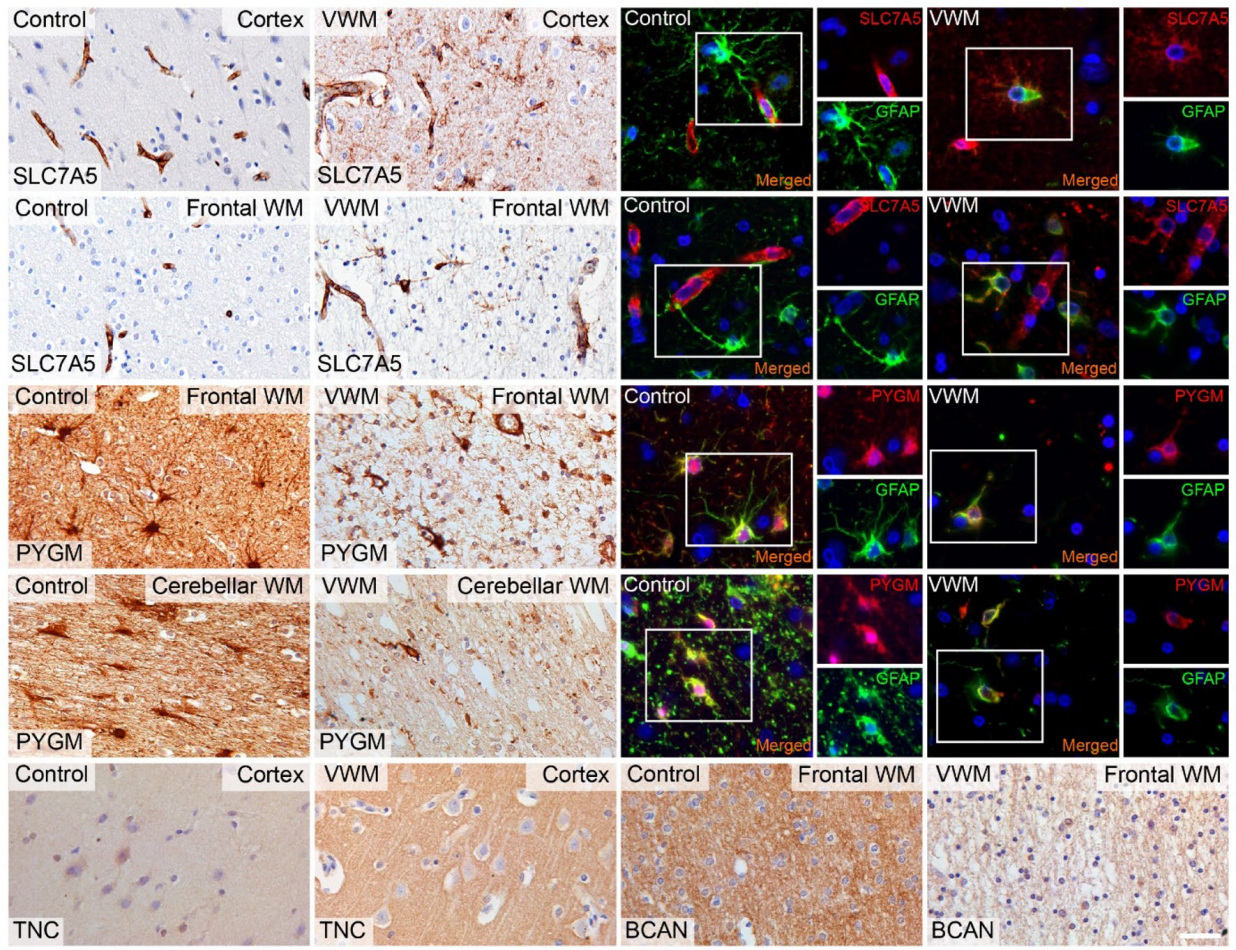
Validation of selected dysregulated proteins in brain tissue of controls and VWM patients. Immunohistochemical stains against SLC7A5, PYGM, TNC, and BCAN confirms differential expression in selected brain regions consistent with proteomics findings. Double labeling with GFAP confirms cellular expression of SLC7A5 and PYGM in GFAP^+^ astrocytes. Scale bar: 40 µm

Immunohistochemical analysis of selected dysregulated proteins confirmed altered expression and involved structural components in affected brain regions of VWM patient tissue at disease end-stage versus controls (Fig. 9). Only a few of these proteins displayed a monotonic behavior. Notably, TNC and BCAN were expressed in the extracellular matrix, while expression of SLC7A5 and PYGM was localized in cells especially in VWM patients. Double labeling with glial fibrillary acidic protein (GFAP) identified these cells as astrocytes (Fig. 9). Expression of a selection of these dysregulated proteins (TNC and BCAN) was further validated on *2b5*^*ho*^ mouse tissue (Supplementary Fig. 3), showing differential expression over time consistent with proteomics findings. Taken together, proteins were concordantly dysregulated in both mouse and human at disease end-stage. A majority of the differentially expressed proteins were either specific to *2b5*^*ho*^ mice or VWM patients (Fig. 8a–f; Supplementary Table 5), suggesting that on protein level *2b5*^*ho*^ mice only partially reflect VWM disease in patients.

## Discussion

Analysis of post mortem tissue allows understanding the pathology of a disease at end-stage. In VWM patients at disease end-stage, the brain white matter is severely affected, with telencephalic areas being more damaged than cerebellar and brainstem areas. Differences in regional susceptibility is also observed within telencephalic areas, with the cerebral hemispheric white matter being more severely affected than other areas like the corpus callosum [3, 11]. Although predominantly a white matter disorder, VWM also affects the gray matter [15]. Neuropathology typically includes meagre reactive gliosis, paucity of myelin, abnormal axons, and immature astrocytes and oligodendrocytes [2–4, 10–14]. Recent studies showed that alterations in proteins involved in cellular metabolism also play a role in VWM [15, 16, 25–28]. It, however, remains unclear which molecular changes contribute to its pathogenesis. Here, we aimed at gaining more insight into the molecular basis of VWM disease progression through the *2b5*^*ho*^ mouse model of VWM. We describe the temporal proteome of disease-relevant brain regions in the *2b5*^*ho*^ mice and focused on proteins that consistently change over time, assuming that these changes are associated with disease development and progression. We then assessed the commonalities in protein changes between *2b5*^*ho*^ mouse and VWM patient brains to define alterations relevant to the human disease.

We found that an increase in proteins involved in amino acid metabolism and transport is an early molecular feature of VWM and plays a role through the disease course in the cerebellum and cortex, but not in the other examined regions. Notably, a majority of protein changes observed early in pathology development does not display a monotonic behavior. This indicates that early molecular alterations in the *2b5*^*ho*^ brain, especially in the cerebellum and cortex, contribute to the disease, but may not necessarily drive the disease progression. Alterations in amino acid metabolism and transport have been implicated in VWM before. For example, increased levels of amino acids have been reported in the *2b5*^*ho*^ mouse brain [29]. Importantly, similar changes were found in the brain and cerebrospinal fluid of VWM patients [16, 30]. Earlier studies also showed a dysregulation in amino acid transport in the *2b5*^*ho*^ mouse brain at transcriptome and proteome levels [9, 24]. It is speculated that changes in amino acid-related processes in VWM are linked to ISR dysregulation [9, 24]. Thus, our data suggest that dysregulation of the ISR occurs early in VWM pathogenesis, which is in agreement with a previous study [9]. To further confirm this, we compared the present work with an independent proteomic dataset of *2b5*^*ho*^ mice treated with an ISR inhibitor to identify relevant ISR targets and assess how these targets change in different brain regions over time at the protein level [24]. We identified overlapping ISR targets between the two datasets. Importantly, dysregulation of some of these ISR targets already occurs at 1 month of age in selected brain regions. This indicates that dysregulation of this cellular stress response starts before clinical disease onset. The results, however, should be interpreted with caution, since only a few overlapping ISR targets were found between the two proteomic datasets. This could in part be explained by differences in study design. Wong et al. [24] only analyzed the *2b5*^*ho*^ mouse proteome of a single region (the cerebellum) at one age (7 months), while the present work studied four differently affected brain regions of *2b5*^*ho*^ mice at different ages. Future studies analyzing the regional brain proteome changes in *2b5*^*ho*^ mice after ISR inhibitor treatment may further help us confirm our results.

It remains unclear why other brain regions are relatively unaffected at the protein level in early disease stages in *2b5*^*ho*^ mice; this may in part be explained by regional differences in susceptibility to the defect in eIF2B. Proteins associated with the subunits of the eIF2B complex (eIF2Bβ, eIF2Bδ) were already downregulated in the cerebellum and cortex of *2b5*^*ho*^ mice at 1 month of age before clinical onset. By contrast, eIF2B complex-related proteins were downregulated in the corpus callosum of *2b5*^*ho*^ mice only starting at clinical onset at 4 months of age, whereas in the brainstem, these proteins only decreased in expression at 4 months and were unaffected at later disease stages. On the basis of these findings, we speculate that brain regions are affected at different disease stages in the *2b5*^*ho*^ mice, with the brainstem being the least affected throughout the disease course.

Indeed, a substantial number of protein changes occurred following clinical symptom onset across all brain regions except the brainstem. These changes persisted throughout disease progression. Remarkably, only a small proportion of proteins were functionally annotated in the cerebellum and cortex, indicating that the majority of protein changes in these regions do not reflect known alterations in specific biological processes or cellular components. By contrast, in the corpus callosum, we observed that many proteins involved in response to stress and oligodendrocyte differentiation were downregulated. These are processes previously associated with human VWM [9, 11, 14, 31]. We now add to earlier findings and show that they are affected in the *2b5*^*ho*^ corpus callosum following symptom onset, and persist as the disease progresses. Notably, some of the protein changes identified at this disease stage showed a monotonic decrease in expression, suggesting that these also contribute to disease progression.

We also found protein changes in the *2b5*^*ho*^ mouse cerebellum, cortex, and corpus callosum starting later in the disease process, when mice readily display pronounced VWM-associated pathological features and motor deficits [12]. These changes were present until the end of the *2b5*^*ho*^ mice lifespan. Again, proteome changes found in the cerebellum and cortex belonged to only a few biological processes, including benzene-containing compound metabolic process, regulation of vascular endothelial growth factor production, and positive regulation of mitotic cell cycle. Protein changes in the corpus callosum reflected decrease in proteins involved in the regulation of cytokine production or the extracellular region. Changes in the extracellular matrix have been implicated in VWM before [12, 32]. A previous study showed high levels of hyaluronic acid, a major component of the extracellular matrix, co-varying with severity in affected regions of VWM patients [32]. Importantly, accumulation of this component in VWM is associated with defects in glia maturation, particularly astrocyte and oligodendrocyte [12, 32]. Taken together, proteome changes also occur late in the disease process across all regions except the brainstem, with some reflecting alterations in features associated with VWM. Taking in consideration that these changes present later in the disease process, they could rather be secondary changes as a result of disease progression. Importantly, a majority of these proteins display a monotonic decrease in expression, and could play a role in driving the disease to its end-stage or reflect end-stage disease.

So far, our findings show that the proteome in the brain of *2b5*^*ho*^ mice is affected in a region-specific and time-dependent manner. Importantly, some proteome changes reflected features well-known in VWM, indicating that this mouse model recapitulates aspects of the human disease. It, however, remained unclear whether protein changes in *2b5*^*ho*^ mice are the same as in patients. To this end, we compared protein changes detected in mouse and human brain regions on the basis of their regional vulnerability in VWM at disease end-stage. We found that proteome changes are similar between *2b5*^*ho*^ mouse and VWM patient brains to a considerable extent. In the *2b5*^*ho*^ mice, the cerebellum and corpus callosum are affected, but not cystic [12]. Based on this, we compared these mouse brain regions with the cerebellar white matter of VWM patients, a region that also shows no cystic degeneration [3]. Our comparative analysis, however, revealed only a few concordantly altered proteins here. Remarkably, protein changes in the *2b5*^*ho*^ cerebellum and corpus callosum shared more commonalities with the frontal white matter of VWM patients. This suggests that protein changes in these mouse brain regions recapitulate proteome changes observed in the frontal white matter better than in the cerebellar white matter of VWM patients. Thus, the *2b5*^*ho*^ mouse model partly reflects the disease in VWM patients at the protein level, with proteome changes in the cerebellum/corpus callosum and cortex of *2b5*^*ho*^ mice showing similarities to those found in the cerebellar and frontal white matter and cortex of VWM patients, respectively. By contrast, proteome changes in the brainstem of *2b5*^*ho*^ mice did not show any similarities with the pons white matter of VWM patients. This is of no surprise, since initial protein changes detected following symptom onset in the mouse brainstem were only transient. Consistent with this, a recent study showed that magnetic resonance imaging (MRI) signal abnormalities occur in the brainstem of VWM patients upon stress, and that they may improve or even resolve over time [33]. This suggests the presence of a possible compensatory mechanism in the brainstem throughout the disease course in *2b5*^*ho*^ mice and VWM patients.

Based on this, we propose that this proteome resource could aid in understanding part of the pathogenesis underlying VWM by highlighting not only early versus late disease alterations, but also potential molecular drivers of disease progression. We define candidate molecular drivers of disease progression as proteins showing a consistent change—either up- or downregulated—in expression level as the disease progresses. For example, there are changes in expression of proteins involved in oligodendrocyte differentiation in the cortex of VWM patients. One of these peptidylarginine deiminase type-2 (PADI2), was markedly reduced in expression also in *2b5*^*ho*^ mice. Peptidylarginine deiminases convert arginine residues to citrullines (also known as citrullination) [34]. PADI2 is predominantly found in glia, particularly oligodendrocytes [35]. It has a dual role in both oligodendrocyte differentiation and myelination [36]. Interestingly, both PADI2 deficiency and overexpression result in defects in myelin compaction and stability [36, 37], suggesting that a balanced PADI2 activity is crucial. Dysregulated PADI2 activity has been implicated in several neurodegenerative diseases, including multiple sclerosis [38]. We hypothesize that this protein could play a similar role in VWM, and dysregulate myelin deposition and maintenance as well as oligodendrocyte differentiation. In the *2b5*^*ho*^ mouse cortex, PADI2 expression level is affected late in the disease course and shows a monotonic decrease as the disease progresses. On the basis of these findings, we postulate that PADI2 expression is also dysregulated after clinical symptom onset in the cortex of VWM patients, and thus may play a role through the course of the disease. Other differentially expressed proteins in the VWM mouse and patient cortex, such as disheveled associated activator of morphogenesis-2 (DAAM2) and tenascin-C (TNC), are also associated with oligodendrocyte differentiation [39, 40].

Additionally, we found a reduction in the expression of proteins related to cellular metabolism in the cerebellar white matter of VWM patients. Amongst these were glycogen phosphorylase (PYGM) and serine hydroxymethyltransferase 2 (SHMT2), which are proteins involved in glycogenolysis [41] and glycine metabolism [42], respectively. In *2b5*^*ho*^ mouse cerebellum and corpus callosum, PYGM and SHMT2 displayed decrease and increase in expression level, respectively, either following clinical symptom onset or later in the disease process. Similar to PADI2, it can be speculated that these proteins, or aspects of their cellular metabolism, are already dysregulated at clinical symptom onset in the cerebellar white matter of VWM patients and contribute to disease progression.

Assessment of the expression of selected dysregulated proteins in VWM patient tissue by immunohistochemistry confirms dysregulation of these proteins, and reveals in which structural components the altered protein expression occurs. PYGM expression in the frontal and cerebellar WM of VWM patients, for example, is decreased in the neuropil, but relatively maintained in astrocytic cell bodies and their blunt processes. By contrast, immunostaining against the amino acid transporter SLC7A5 shows preserved expression in vascular endothelial cells but aberrant expression in astrocytes in the cortex and frontal WM, which can explain the total upregulation of this protein at the proteome level. Regarding proteins belonging to the extracellular matrix, such as TNC and brevican (BCAN), immunohistochemistry also confirms the proteomics findings.

Mouse brain lacks cerebral white matter, hampering a comparison to the VWM patient frontal white matter. Remarkably, however, we found that the proteome of the *2b5*^*ho*^ cerebellum and corpus callosum shows considerable overlap in protein changes with the frontal white matter of VWM patients. This was surprising, as the human neuropathology of these regions differs, with the frontal white matter undergoing cystic degeneration and cavitation, while the white matter of cerebellum and corpus callosum does not. We could speculate that the proteome similarities between the VWM mouse corpus callosum and cerebellum and the patient frontal white matter reflect the disease mechanisms occurring before tissue cavitation. Identification of these proteins at onset and during disease progression in *2b5*^*ho*^ mice, and their confirmation in VWM patients has important implications. We could assume that their differential expression over time is similar in mice and patients. Further in vivo studies are needed to verify this, including their assessment on cerebrospinal fluid and perhaps peripheral blood. If this was the case, these proteins could be used as biomarkers to help monitor disease activity also during therapeutic clinical trials.

Some limitations apply to this work. The *2b5*^*ho*^ mouse model mimics disease in VWM patients at the protein level only to a certain extent. Although some dysregulated proteins in the *2b5*^*ho*^ mouse brains are concordantly altered in VWM patient brains, we observed that a majority of protein changes were either specific to mouse or human, indicating that this mouse model does not completely recapitulate human VWM. This may in part be explained by interspecies differences, including differences in lifespan, and lack of cerebral white matter in the mouse brain. Given these differences, it is possible that relevant pathophysiological features are not conserved between the species. Another possibility is that the *2b5*^*ho*^ mouse model only represents some aspects of the human disease at the protein level. Although some protein changes in the *2b5*^*ho*^ brain reflect alterations in specific processes previously associated with human VWM, specific differentially expressed proteins are distinct between mice and patients. This suggests that, at protein level, the *2b5*^*ho*^ mouse and VWM patients share involvement of the same molecular processes, but not the differential expression of the single proteins driving these processes. This also indicates that other VWM disease models should be taken under consideration to study VWM pathogenesis, as these may represent other aspects of VWM that are not recapitulated in *2b5*^*ho*^ mice.

Taken together, our data revealed protein changes in the *2b5*^*ho*^ mouse brain associated with early to late disease stages and those that, in the mouse, drive disease progression. Based on our comparative analysis, we suggest that the spatiotemporal proteome profile of the *2b5*^*ho*^ mouse brain may aid as a resource to gain insight into a fraction of the pathogenesis of VWM. No single disease model, however, is a perfect representation of the human disease. Future studies are therefore warranted to thoroughly characterize each available VWM disease model to determine which model to employ for a given research question.

### Supplementary Information

Below is the link to the electronic supplementary material.

Supplementary Table 1. Demographic features of controls and genetically proven VWM patients used for proteome analysis of the white matter in the cerebellum. (XLSX 10 KB)

Supplementary Table 2. List of proteins with quantitative values in all samples. WT Wild Type, Cb Cerebellum, Cc Corpus callosum, Ctx Cortex, Bs Brainstem. (XLSX 9125 KB)

Supplementary Table 3. Proteins differentially expressed in cerebellum, corpus callosum, cortex and brainstem of *2b5*^*ho*^ vs. wild type mice of 1, 4, 7, and 12 months of age. (XLSX 4082 KB)

Supplementary Table 4. Gene ontology analysis of proteins differentially expressed in cerebellum, corpus callosum, and cortex of *2b5*^*ho*^ mice starting from 1, 4, and 7 months of age. (XLSX 23 KB)

Supplementary Table 5. Comparisons of VWM patient and *2b5*^*ho*^ mouse brain regional proteomes. (XLSX 34 KB)

**Supplementary Fig. 1 Spatiotemporal protein expressions in WT and** ***2b5***^***ho***^ **mice**. Heatmaps showing Pearson correlation between individual samples from the cerebellum, corpus callosum, cortex and brainstem of WT and *2b5*^*ho*^ mice at 1, 4, 7, and 12 months of age. (PNG 479 KB)

**Supplementary Fig. 2 Global protein expression changes between WT and** ***2b5***^***ho***^ **mice at different ages**. Volcano plots displaying the significant protein expression changes between WT vs. *2b5*^*ho*^ mice in the cerebellum, corpus callosum, cortex, and brainstem at 1, 4, 7, and 12 months. Significantly down- and upregulated proteins (|log2FC| > 1, adj. p < 0.05) in the *2b5*^*ho*^ mice are highlighted in blue (left) and red (right), respectively. Proteins not significantly altered are highlighted in grey. (PNG 409 KB)

**Supplementary Fig. 3 Validation of selected dysregulated proteins in the brain regions of interest in WT and** ***2b5***^***ho***^ **mice**. Immunohistochemical stains against TNC and BCAN in the cortex and corpus callosum of WT and *2b5*^*ho*^ mice at different ages, respectively. Stains confirm differential expression in the selected brain regions over time consistent with proteomics findings. Scale bar: 20 µm. (PNG 5670 KB)

## Data Availability

Proteomics data have been deposited into the ProteomeXchange Consortium via the PRIDE partner repository with the dataset identifier PXD045041 (human cerebellar white matter proteomic data) and PXD043872 (mouse brain regional proteomic data) [43, 44].

## Abbreviations

ACN: Acetonitrile
Adj.: Adjusted
ALDH18A1: Aldehyde dehydrogenase family 18 member A1
APOE: Apolipoprotein E
AQP4: Aquaporin-4
ASNS: Asparagine synthetase
BCAN: Brevican core protein
BH: Benjamini–Hochberg
Bs: Brainstem
BTBD17: BTB/POZ domain-containing protein 17
CAA: Chloroacetamide
CARS1: Cysteine—tRNA ligase, cytoplasmic
Cb: Cerebellum
Cc: Corpus callosum
CLU: Clusterin
CST3: Cystatin-C
CTH: Cystathionine gamma-lyase
Ctx: Cortex
DAAM2: Disheveled-associated activator of morphogenesis 2
DIA: Data-independent acquisition
eIF2: Eukaryotic translation factor 2
eIF2Bε: Translation initiation factor eIF-2B subunit epsilon
eIF2B: Eukaryotic translation initiation factor 2B
eIF2Bβ: Translation initiation factor eIF-2B subunit beta
eIF2Bδ: Translation initiation factor eIF-2B subunit delta
ENTPD2: Ectonucleoside triphosphate diphosphohydrolase 2
EPHX2: Bifunctional epoxide hydrolase 2
EPRS1: Bifunctional glutamate/proline—tRNA ligase
FA: Formic acid
FC: Fold change
FDR: False discovery rate
GFAP: Glial fibrillary acidic protein
GPRC5B: G-protein coupled receptor family C group 5 member B
GSTM1: Glutathione *S*-transferase Mu 1
GSTM5: Glutathione *S*-transferase Mu 5
HPX: Hemopexin
ISR: Integrated stress response
MS: Mass spectrometry
PADI2: Protein-arginine deiminase type-2
PCK2: Phosphoenolpyruvate carboxykinase [GTP], mitochondrial
PHGDH: Phosphoglycerate dehydrogenase
PLPP3: Phospholipid phosphatase 3
PSAT1: Phosphoserine aminotransferase
PSPH: Phosphoserine phosphatase
PYGM: Glycogen phosphorylase, muscle form
S100A13: Protein S100-A13
S100A16: Protein S100-A16
SHMT2: Serine hydroxymethyltransferase, mitochondrial
SLC3A2: 4F2 cell-surface antigen heavy chain
SLC7A5: Large neutral amino acids transporter small subunit 1
SPARCl1: SPARC-like protein 1
SPEED: Easy extraction and digestion
TCEP: Tris(2-carboxyethyl)phosphine
TFA: Trifluoroacetid acid
TNC: Tenascin
TRIB2: Tribbles homolog 2
VCAM1: Vascular cell adhesion protein 1
VWM: Vanishing white matter
WM: White matter
WT: Wild-type
