# Supplementary figures and images for: Proteomic dissection of vanishing white matter pathogenesis
Source: Cell Mol Life Sci. 2024 May 24;81(1):234. doi: 10.1007/s00018-024-05258-4 (PMC11126554; doi:10.1007/s00018-024-05258-4)

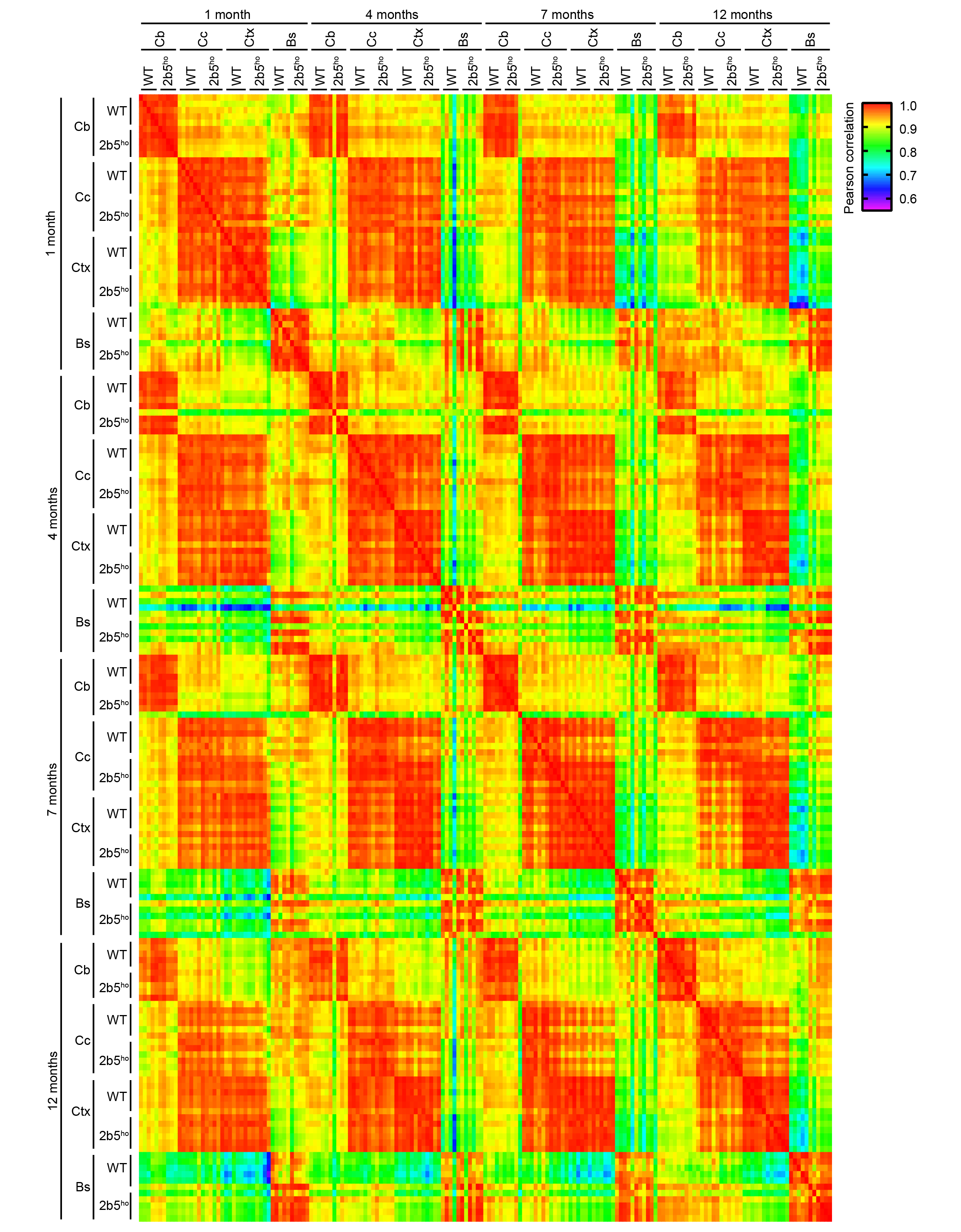

Supplement: Supplementary file 6 — Supplementary Fig. 1 Spatiotemporal protein expressions in WT and 2b5ho mice. Heatmaps showing Pearson correlation between individual samples from the cerebellum, corpus callosum, cortex and brainstem of WT and 2b5ho mice at 1, 4, 7, and 12 months of age. (PNG 479 KB) [file 18_2024_5258_MOESM6_ESM.png]

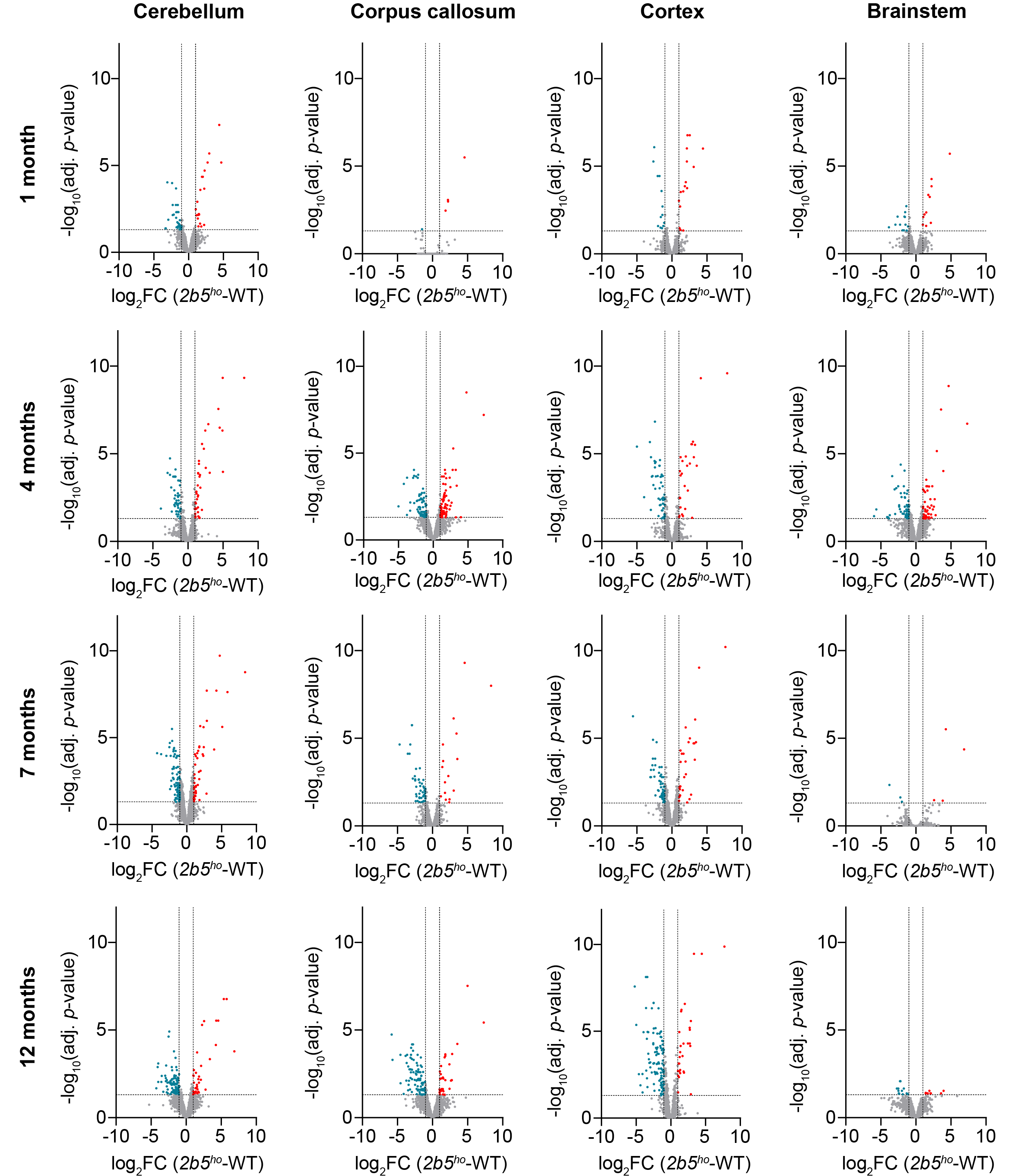

Supplement: Supplementary file 7 — Supplementary Fig. 2 Global protein expression changes between WT and 2b5ho mice at different ages. Volcano plots displaying the significant protein expression changes between WT vs. 2b5ho mice in the cerebellum, corpus callosum, cortex, and brainstem at 1, 4, 7, and 12 months. Significantly down- and upregulated proteins (|log2FC| > 1, adj. p < 0.05) in the 2b5ho mice are highlighted in blue (left) and red (right), respectively. Proteins not significantly altered are highlighted in grey. (PNG 409 KB) [file 18_2024_5258_MOESM7_ESM.png]

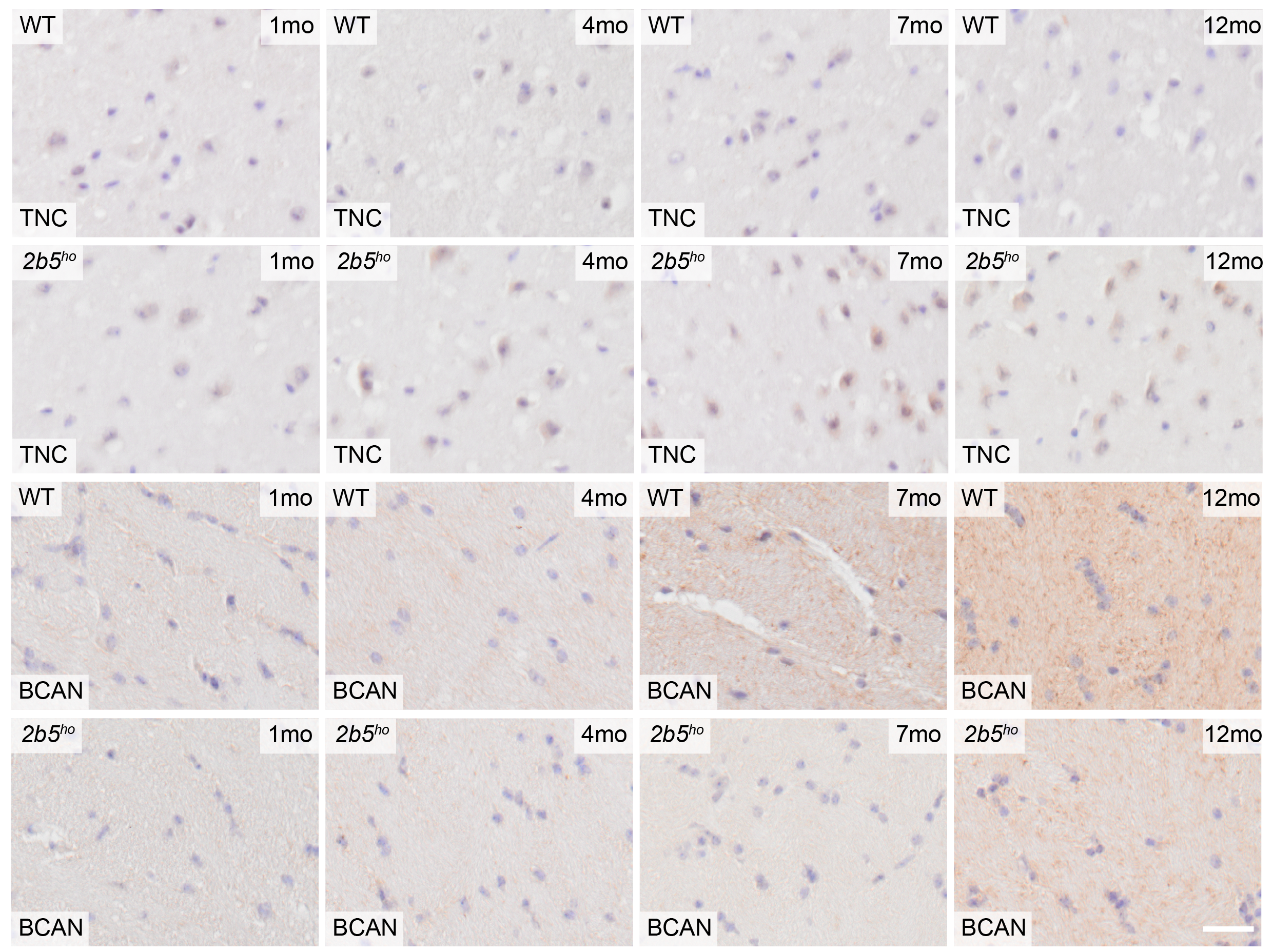

Supplement: Supplementary file 8 — Supplementary Fig. 3 Validation of selected dysregulated proteins in the brain regions of interest in WT and 2b5ho mice. Immunohistochemical stains against TNC and BCAN in the cortex and corpus callosum of WT and 2b5ho mice at different ages, respectively. Stains confirm differential expression in the selected brain regions over time consistent with proteomics findings. Scale bar: 20 µm. (PNG 5670 KB) [file 18_2024_5258_MOESM8_ESM.png]
